# Season-long changes in COD performance, jump, and flexibility in elite youth soccer players

**DOI:** 10.3389/fspor.2026.1751542

**Published:** 2026-02-20

**Authors:** Michelangelo Palco, Gabriele Giuca, Alessandro Giorgio, Danilo Leonetti, Roberto Simonetta, Filippo Familiari

**Affiliations:** 1 Department of Orthopaedic and Trauma Surgery, Casa di Cura Caminiti, Villa San Giovanni, Italy; 2 Department of Biomedical Sciences and Morphological and Functional Images, Section of Orthopaedics and Traumatology, University of Messina, Messina, Italy; 3 Department of Orthopaedic and Trauma Surgery and Research Center on Musculoskeletal Health, Magna Graecia University, Catanzaro, Italy

**Keywords:** change-of-direction, longitudinal, maturation, sit-and-reach, standing broad jump, youth soccer

## Abstract

**Background:**

In elite youth soccer, the interplay between the development of key physical qualities, change-of-direction (COD) performance, explosive strength, and flexibility, remains poorly understood. Conventional training often assumes transfer effects, yet empirical evidence on how these qualities co-occur within elite youth cohorts across a competitive season remains limited. We tested the hypothesis that flexibility and explosive strength would be positively associated with COD performance at end-season (T2).

**Methods:**

Forty-five elite male youth players (Serie A academy; U12–U14) were monitored across one competitive season. COD performance (T-Test completion time), flexibility (sit-and-reach), and explosive strength (standing broad jump, SBJ) were assessed at the season's start (T0; September 1, 2024) and end (T2; May 1, 2025). Primary analyses used paired *t*-tests with Cohen's d_z and 95% confidence intervals (CI). Pearson correlations (with 95% CI) were computed cross-sectionally at T2.

**Results:**

Significant season-long improvements were observed in flexibility [Δsit-and-reach = + 1.83 ± 1.42 cm; 95% CI (1.40, 2.26); *p* = 4.87 × 10^−11^; d_z = 1.29], explosive strength [SBJ Δ =  + 12.8 ± 3.8 cm; 95% CI (11.66, 13.94); *p* < 1 × 10^−20^; d_z = 3.37], and COD performance [Δ*T*-Test = −1.16 ± 0.87 s; 95% CI (−1.42, −0.90); *p* = 1.86 × 10^−11^; d_z = −1.33]. At T2, SBJ was moderately associated with COD performance [SBJ vs. *T*-Test: r = −0.41, *p* = 0.005; 95% CI (−0.63, −0.13)] and with flexibility [SBJ vs. sit-and-reach: r = 0.32, *p* = 0.032; 95% CI (0.03, 0.56)], whereas sit-and-reach was not significantly related to *T*-Test time (r = −0.15, *p* = 0.31).

**Conclusions:**

Elite youth players improved COD performance, flexibility, and horizontal power across the season. Cross-sectionally, higher SBJ performance co-occurred with faster COD performance at end-season, while sit-and-reach flexibility did not. These findings support multi-domain monitoring and targeted training prescriptions, while highlighting the need for maturation-informed designs to test longitudinal coupling of individual adaptations.

## 1 Introduction

Soccer, as a high-intensity intermittent sport, imposes multifaceted physiological demands on athletes, requiring seamless coordination of technical, tactical, and physical attributes for optimal performance (1). At the elite youth level, where players transition from foundational skill acquisition to competitive proficiency, physical capacities such as joint mobility, explosive strength, and aerobic capacity form the bedrock of athletic longevity and injury resilience (1). Joint mobility, encompassing range of motion (ROM) in key lower-limb articulations like the hip, knee, and ankle, facilitates efficient biomechanical execution during sprints, directional changes, and movements ubiquitous in match play (2). Deficits in mobility not only constrain force transmission but also elevate strain on musculoskeletal tissues, predisposing young athletes to overuse injuries that can derail developmental trajectories (3).

Explosive strength, manifested as the rapid generation of force through the stretch-shortening cycle (SSC), underpins soccer-specific actions including acceleration, jumping, and shooting (4). This neuromuscular quality is particularly pivotal in youth cohorts, where maturation-induced variability in muscle architecture and neural drive can amplify performance disparities (5). Concurrently, aerobic fitness supports repeated high-intensity efforts and recovery during match play, influencing fatigue resistance and the ability to express technical actions under load (6, 7). However, the present study focuses on three field-based qualities commonly monitored in academies, COD performance, horizontal power, and posterior-chain flexibility because these domains are frequently targeted within integrated in-season conditioning and may exhibit non-uniform adaptation patterns during growth (7–13).

Plyometric training emerges as a promising bridge, leveraging SSC to concurrently bolster explosive strength and mobility via eccentric loading that enhances tendon stiffness and ROM (14). In youth settings, typical in-season plyometric dosing comprises 2–3 sessions/week with ∼60–120 ground contacts/session for 6–10 weeks, progressed from extensive to intensive drills under qualified supervision. Meta-analyses confirm moderate gains in jump height (ES = 0.6) and sprint speed among adolescents, with added benefits for agility when integrated with dynamic stretching (15, 16). Dynamic stretching, prepares neuromuscular function and range of motion; injury effects are context-specific and not uniform (17). However, initial implementation may provoke transient performance dips via acute fatigue and neural recalibration hallmarks of supercompensation that resolve within 4–6 weeks (18).

This interplay challenges prevailing paradigms, which prioritize volume over integration, often overlooking youth-specific maturation confounds like peak height velocity (PHV) (19). During PHV, explosive gains lag behind aerobic developments unless targeted, as longitudinal tracking in elite academies reveals maturity-modulated divergences (20). Herein lies the novelty: while cross-sectional snapshots abound (21), our longitudinal design captures seasonal flux in a homogeneous elite cohort, hypothesizing that improvements in flexibility and explosive strength would positively correlate with changes in COD performance, with training-induced synergies reflected in parallel improvements. By elucidating these dynamics, we offer practical insight for integrated youth training in elite academies, while future controlled studies will be needed before informing broader policy (22).

At the applied academy level, season-long monitoring is especially relevant because adolescent players represent a large global population and carry a meaningful burden of time-loss injuries and performance variability during growth (23, 24). However, longitudinal evidence on whether within-player changes in flexibility and explosive strength track with changes in COD performance across a full competitive season remains limited in elite youth cohorts (13). Clarifying these co-development patterns can inform monitoring strategies and the design of integrated, but still specifically targeted, training prescriptions (25).

The aims of this study were to: (i) quantify season-long changes in flexibility (sit-and-reach), explosive strength (standing broad jump), and COD performance (*T*-Test completion time) in elite youth soccer players; (ii) examine cross-sectional (T2) associations among these qualities; and (iii) discuss implications for applied academy monitoring and training. We hypothesized that better explosive strength and flexibility would be associated with better COD performance at T2.

## 2 Materials and methods

### 2.1 Ethical considerations and participant recruitment

This prospective longitudinal study adhered to the Declaration of Helsinki and received institutional review board approval by Technical and Scientific Committee at the Department of Medicine and Health Science of the University of Molise (Italy), Protocol 23/2022, on November 11th, 2022. Parental consent and child assent were obtained. Fifty elite male youth soccer players (Serie A academy teams competing in national U12–U14 leagues) were recruited from Professional Football's academy in September 2024. Inclusion criteria: active roster status, no musculoskeletal injuries in prior 6 months, and medical clearance. Exclusion: chronic conditions or non-compliance with testing.

Five participants withdrew prior to follow-up due to relocation or incomplete attendance, yielding 45 completers (all male). Based on available birth-date records (44/45), the cohort's chronological age at baseline (T0) was 11.7 ± 0.5 years (range: 10.8–12.6). Participants were retrospectively classified according to the Participant Classification Framework proposed by McKay et al. (26); given their full-time academy status and structured multi-session weekly training, the cohort is best described as Tier 3 (highly trained/national-level developmental athletes). No adverse events occurred during testing. To mitigate maturation bias, testing spanned one competitive season (September 2024-May 2025), capturing pre- and post-influences of a standardized program blending technical (60%), tactical (20%), and physical (20%) sessions: 3–5 weekly, 90–120 min each. Physical components incorporated progressive pliometrics and dynamic warm-ups, per academy protocols, without altering for study purposes. Players trained within a tightly defined U12–U14 competitive band under a standardized academy program. Biological maturation (e.g., maturity offset/peak height velocity) was not directly assessed because these markers were not routinely collected within the academy monitoring system during the study period. This limitation is explicitly considered when interpreting both performance changes and association analyses.

### 2.2 Anthropometric assessments

Across the competitive season, the academy followed a typical 3–5 sessions/week microcycle (90–120 min per session) plus one weekend match. The weekly content comprised approximately ∼60% technical, ∼20% tactical, and ∼20% physical work. Physical components included dynamic warm-ups, progressively dosed plyometrics (integrated within practices), and COD drills aligned with age-group objectives; no training content was altered for study purposes. Measurements preceded physical tests each session, conducted by certified anthropometrists using calibrated stadiometers (Seca 213, Hamburg, Germany) and digital scales (Tanita BC-418, Tokyo, Japan). Height was recorded to 0.1 cm (barefoot, heels together, maximal inhalation); weight to 0.1 kg (light clothing, post-voiding). Assessments occurred at baseline (September 1, 2024) and follow-up (May 1, 2025) under thermoneutral conditions (20–22 °C, 50%–60% humidity). Participants ab-stained from caffeine 24 h and strenuous training 48 h prior to testing; euhydration was encouraged.

### 2.3 Physical performance testing overview

All assessments were administered by the same two experienced examiners, in the same facility and afternoon time window (15:00–18:00). A standardized testing battery was conducted at the beginning of the season (T0; September 1, 2024) and at the end of the season (T2; May 1, 2025). The tests administered at both T0 and T2 were performed in the following order: anthropometrics → sit-and-reach → standing broad jump (SBJ) → COD (*T*-Test). Measurement quality was addressed through strict standardization (same examiners, same facility and time window, fixed test order, multiple trials, and predefined criteria for invalid attempts). While a dedicated in-season test–retest session was not feasible within this academy schedule, the sit-and-reach, SBJ, and *T*-Test have demonstrated acceptable-to-excellent reliability and validity in youth field-based settings in previous work, supporting their use for longitudinal monitoring when standardized protocols are applied (27–32).

### 2.4 Flexibility: sit and reach test

Flexibility was assessed using the sit-and-reach test, which provides a practical estimate of hamstring–lumbar flexibility in youth populations. Participants sat barefoot with knees fully extended and the soles of the feet placed flat against a standardized sit-and-reach box. With hands overlapped and arms extended, they slowly reached forward along the measuring scale without ballistic movement, maintaining maximal reach for ∼2 s. Players performed three trials per test. For the sit-and-reach and SBJ, trials were separated by ∼30–60 s of recovery; for the *T*-test, trials were separated by 60 s. A standardized 10–15 min passive recovery separated the different tests. As a practical field test, sit-and-reach provides a global estimate of posterior-chain flexibility and does not capture joint-specific ROM; this constraint is acknowledged in the Limitations (27, 28). The best value (cm) across trials was retained for analysis.

### 2.5 Explosive strength: standing broad jump (SBJ)

From a shoulder-width stance behind a take-off line, players performed a two-footed horizontal jump with natural arm swing; the best of three attempts was recorded as the horizontal distance (cm) from the line to the posterior heel landing mark, using a measuring tape on the turf. Rest between trials: 30–60 s. The standing broad jump has shown acceptable-to-excellent test–retest reliability in youth cohorts (commonly reported ICCs ≳ 0.90 under standardized field protocols) (28, 29). To mitigate technical error, examiners enforced standardized instructions and repeated any invalid trials (e.g., premature step, heel-mark uncertainty).

### 2.6 Change-of-direction (COD): *T*-test

COD speed was assessed using the *T*-Test with cones at 9.14 m (forward) and 4.57 m (lateral). Single-beam timing gates (start/finish) recorded the best of three maximal trials, each separated by 60 s. Players sprinted forward, shuffled laterally right/left, returned to center, and backpedaled to the start; lower times indicate faster COD performance (29).

### 2.7 Statistical analyses

Data were processed in Microsoft Excel 365 (Redmond, USA) and verified via Python (v3.12; pandas/scipy). Change scores (Δ = T2−T0) were computed for each outcome. Δ distributions were inspected via Q–Q plots and Shapiro–Wilk and were approximately normal; therefore, paired *t*-tests were used. We report mean differences with 95% confidence intervals and Cohen's d_z (mean Δ/SD Δ). Cross-sectional associations among outcomes at follow-up (T2) were examined using Pearson's r with 95% confidence intervals (Fisher's z). No *a priori* power analysis was performed because sample size was constrained by the fixed academy roster; accordingly, we emphasize effect sizes and confidence intervals. Effect sizes (Cohen's d_z) for paired comparisons were interpreted as small (≈0.20), moderate (≈0.50), and large (≈0.80). Reporting follows best practice for within-subject designs.

## 3 Results

Forty-five participants completed the study, with complete datasets for all variables. No systematic dropouts biased outcomes.

### 3.1 Anthropometric changes

Seasonal growth was robust (Table 1). Body weight increased from 46.7 ± 10.2 kg (range 30.0–64.0; median 46.0; IQR 39.0–53.0) to 48.4 ± 10.5 kg (32.8–65.0; median 47.5; IQR 40.8–54.0), with a mean Δ = + 1.70 kg [≈+3.64%; t (44) = 10.37, *p* = 2.17 × 10^−13^]. Height increased from 149.3 ± 8.7 cm (132.0–167.0; median 149.0; IQR 144.0–156.0) to 152.8 ± 8.9 cm (138.0–170.0; median 152.0; IQR 147.0–159.0), Δ =  + 3.50 cm [≈+2.34%; t(44) = 16.77, *p* = 9.89 × 10^−21^].

**Table 1:** Anthropometric changes from baseline (T0) to end-season (T2) (*n* = 45).

Variable	Baseline (T0) Mean ± SD	Baseline Median [IQR]	End-season (T2) Mean ± SD	End-season Median [IQR]	Baseline Range	End-season Range	Mean Δ (T2−T0)	% Change	t	p
Weight (kg)	46.7 ± 10.2	46.0 [39.0–53.0]	48.4 ± 10.5	47.5 [40.8–54.0]	30.0–64.0	32.8–65.0	+1.70	+3.64	10.37	2.17 × 10^−13^
Height (cm)	149.3 ± 8.7	149.0 [144.0–156.0]	152.8 ± 8.9	152.0 [147.0–159.0]	132.0–167.0	138.0–170.0	+3.50	+2.34	16.77	9.89 × 10^−21^

### 3.2 Changes in physical performance measures

Significant season-long improvements were observed across all performance outcomes (Table 2). Sit-and-reach increased from 3.12 ± 4.21 to 4.95 ± 4.87 cm (Δ =  + 1.83 ± 1.42; *p* = 4.87 × 10^−11^; d_z = 1.29). Standing broad jump distance improved from 179.8 ± 24.3 to 192.6 ± 26.1 cm (Δ =  + 12.8 ± 3.8; *p* < 1 × 10^−2^⁰; d_z = 3.37). COD performance improved, as reflected by a reduction in *T*-Test completion time from 13.84 ± 1.23 to 12.68 ± 1.45 s (Δ = −1.16 ± 0.87; *p* = 1.86 × 10^−11^; d_z = −1.33).

**Table 2:** Season-long changes in physical performance outcomes (*n* = 45).

Variable	Timepoints	N	Baseline mean ± SD	Follow-up mean ± SD	Mean Δ (follow–base) ± SDΔ	95% CI of Δ	% Change	*p*-value	Cohen's d_z
Sit-and-reach (cm)	T0 → T2	45	3.12 ± 4.21	4.95 ± 4.87	+1.83 ± 1.42	[1.40, 2.26]	+58.65%	4.87 × 10^−11^	1.29
Standing broad jump (cm)	T0 → T2	45	179.8 ± 24.3	192.6 ± 26.1	+12.8 ± 3.8	[11.66, 13.94]	+7.12%	< 1 × 10^−20^	3.37
*T*-Test (s)	T0 → T2	45	13.84 ± 1.23	12.68 ± 1.45	−1.16 ± 0.87	[−1.42, −0.90]	−8.38%	1.86 × 10^−11^	−1.33

### 3.3 Correlation analyses

At follow-up (T2), jump performance showed a moderate association with COD performance, with greater jump distance related to faster *T*-Test time (r = −0.41, *p* = 0.005; 95% CI: −0.63 to −0.13). Jump performance also correlated positively with sit-and-reach flexibility (r = 0.32, *p* = 0.032; 95% CI: 0.03–0.56). These cross-sectional relationships indicate that, at end-season, higher horizontal power capacity tends to co-occur with better COD performance and greater flexibility within this cohort (Table 3).

**Table 3:** Cross-sectional correlations at follow-up (T2).

Correlation pair	N	Pearson r	95% CI low	95% CI high	*p*-value	t-statistic
SBJ vs. *T*-Test at T2	45	−0.41	−0.63	−0.13	0.005	−2.98
Sit-and-reach vs. *T*-Test at T2	45	−0.15	−0.43	0.15	0.31	−1.02
Sit-and-reach vs. SBJ at T2	45	0.32	0.03	0.56	0.032	2.21

## 4 Discussion

The observed improvements should be interpreted as the consequence of global training exposure within an elite academy environment rather than being attributed solely to the formal “physical training” component. Across a competitive season, technical drills, small-sided games, and match play impose substantial neuromuscular, mechanical, and metabolic loads that likely contributed to the concurrent development of COD performance, flexibility and explosive power (1, 8). The large reduction in *T*-Test completion time (Δ = −1.16 s, d_z = −1.33, *p* < 0.001) represents not merely statistical significance but a practically meaningful improvement in soccer-relevant COD performance (29). This enhancement may reflect neural adaptations in movement efficiency and force application during directional changes, potentially mediated by the progressive plyometric and COD drills embedded into regular training sessions (14, 16). Similarly, the substantial improvement in standing broad jump performance (Δ =  + 12.8 cm, d_z = 3.37, *p* < 0.001) indicates marked development in horizontal power output, which is crucial for sprint acceleration and jumping actions in soccer (4). The improvement in sit-and-reach performance (Δ =  + 1.83 cm, d_z = 1.29, *p* < 0.001) suggests concurrent adaptations in posterior-chain flexibility, potentially influencing movement economy and/or injury-related determinants (2, 3). Importantly, cross-sectional analyses at end-season showed that higher SBJ performance co-occurred with faster COD performance (r = −0.41) and greater sit-and-reach flexibility (r = 0.32), whereas sit-and-reach was not significantly associated with *T*-Test time (r = −0.15). These patterns are consistent with the principle of training specificity operating at a granular level (10): COD outcomes depend not only on strength and power but also on technical execution, deceleration–reacceleration mechanics, and perceptual-cognitive factors that are not captured by flexibility measures alone (29). Our findings align with contemporary evidence syntheses reporting meaningful improvements in youth athletes following structured plyometric or combined strength-plyometric interventions (30–32). Moreover, inter-individual differences in maturation status and responsiveness may contribute to the observed performance dispersion and correlation structure, even within a narrow chronological age band (5, 19, 20). Finally, the concept of “individual response signatures” suggests that athletes may exhibit heterogeneous adaptation capacities across different physiological systems, supporting the need for integrated monitoring paired with targeted prescriptions rather than assuming uniform transfer effects across qualities (12). Some players might be “high responders” to power training but “low responders” to flexibility interventions, or vice versa, creating the observed pattern of dissociated improvements.

From a methodological perspective, our findings challenge the assumption that change scores in different physical qualities should naturally correlate within individuals following integrated training (10, 12). This has important implications for both research design and practical application: For researchers, our results emphasize the importance of analyzing individual response patterns alongside group means, as the latter can mask important heterogeneity in training adaptations (12). Future studies should incorporate more frequent testing timepoints and consider individual maturity status to better understand the temporal dynamics of these adaptations (19, 20). For practitioners, the dissociation between adaptation pathways supports a training philosophy of “complementary targeting” rather than assumed transfer. Coaches should not presume that improvements in one physical domain will automatically translate to others, even within well-designed integrated programs (10). Instead, they must ensure adequate, distinct attention to each physical quality while recognizing that individual players may require different emphases based on their specific response patterns. While our study provides valuable insights, several limitations warrant consideration. The absence of direct maturity assessment (e.g., via peak height velocity estimation) limits our ability to explore maturation-mediated adaptation patterns (19). Furthermore, the sit-and-reach test, while practical, provides a global measure of flexibility that may not fully capture joint-specific mobility changes relevant to soccer performance (27). In addition, training exposure was described qualitatively (weekly structure), but internal and external load were not quantified (e.g., session-RPE, GPS-derived distances, accelerations, or sprint counts). Therefore, we cannot determine whether inter-individual differences in neuromuscular adaptations were driven by differences in training dose, match exposure, or recovery. Finally, the lack of a non-training control group limits causal inference; observed changes may reflect the combined effects of academy training, match play, and normal growth/maturation during this developmental period. Future research should incorporate more frequent assessment timepoints to track the temporal dynamics of adaptation, include direct measures of biological maturation, and employ more sophisticated statistical approaches (such as latent growth curve modeling) to better understand individual adaptation trajectories. Additionally, integrating biomechanical analyses could help elucidate the technical factors that mediate improvements in COD performance independent of general power development. In conclusion, while elite youth soccer players demonstrably improve across multiple physical domains during a competitive season, the dissociation between individual adaptation magnitudes suggests that these improvements occur through at least partially independent neuromuscular pathways. This understanding should inform more personalized, targeted approaches to physical development in the youth soccer context.

## 5 Conclusions

This study demonstrates that elite youth soccer players achieve substantial improvements in change-of-direction speed, explosive strength, and flexibility across a competitive season through integrated training. However, the critical finding of non-significant correlations between individual improvement magnitudes reveals that these adaptations occur through largely independent pathways. This understanding should inform more sophisticated, targeted approaches to physical development in youth soccer, emphasizing the need for complementary training stimuli rather than relying on assumed transfer effects between physical qualities. Future research should incorporate more frequent assessment timepoints and direct maturity measurements to further elucidate the complex dynamics of athletic development in young soccer players.

## Data Availability

The original contributions presented in the study are included in the article/Supplementary Material, further inquiries can be directed to the corresponding author.
